# Characterization of Sex-Based Dna Methylation Signatures in the Airways During Early Life

**DOI:** 10.1038/s41598-018-23063-5

**Published:** 2018-04-03

**Authors:** Cesar L. Nino, Geovanny F. Perez, Natalia Isaza, Maria J. Gutierrez, Jose L. Gomez, Gustavo Nino

**Affiliations:** 1 Department of Electronics Engineering, Pontificia Universidad Javeriana, Bogota, Colombia; 2 Division of Pulmonary and Sleep Medicine, Children’s National Medical Center, Washington, DC USA; 3 Department of Pediatrics, George Washington University School of Medicine and Health Sciences, Washington, DC USA; 4 Center for Genetic Medicine, Children’s National Medical Center, Washington, DC USA; 5 Division of Neonatology, Children’s National Medical Center, Washington, DC USA; 6 Division of Pediatric Allergy Immunology, Johns Hopkins University School of Medicine, Baltimore, MD USA; 7 Division of Pulmonary, Critical Care and Sleep Medicine, Yale University School of Medicine, New-Haven, CT USA

## Abstract

Human respiratory conditions are largely influenced by the individual’s sex resulting in overall higher risk for males. Sex-based respiratory differences are present at birth suggesting a strong genetic component. Our objective was to characterize early life sex-based genomic signatures determined by variable X-chromosome methylation in the airways. We compared male versus female genome-wide DNA methylation in nasal airway samples from newborns and infants aged 1–6 months (N = 12). We analyzed methylation signals across CpG sites mapped to each X-linked gene using an unsupervised classifier (principal components) followed by an internal evaluation and an exhaustive cross-validation. Results were validated in an independent population of children (N = 72) following the same algorithm. X-linked genes with significant sex-based differential methylation in the nasal airway of infants represented only about 50% of the unique protein coding transcripts. X-linked genes without significant sex-based differential methylation included genes with evidence of escaping X-inactivation and female-biased airway expression. These genes showed similar methylation patterns in males and females suggesting unbalanced X-chromosome dosage. In conclusion, we identified that the human airways have already sex-based DNA methylation signatures at birth. These early airway epigenomic marks may determine sex-based respiratory phenotypes and overall predisposition to develop respiratory disorders later in life.

## Introduction

The epidemiology of human respiratory conditions is largely influenced by the individual’s sex. Neonatal respiratory distress, bronchopulmonary dysplasia, viral bronchiolitis, pneumonia, laryngotracheobronchitis (croup) and childhood asthma are all more common in males^1–5^, providing solid evidence that males are more prone to respiratory disorders than females, particularly during early life^1–5^. Gender disparities in respiratory disease are partially explained by hormonal, anatomical and behavioral factors^1^. However, genetic and genomic factors associated with sex determination, including differential gene methylation and resulting changes in gene expression, are potential contributors to these disparities.

The most important genetic sex-based difference across species is the presence of an additional copy of the X-chromosome in females (XX) relative to males (XY)^6^. In humans the Y chromosome contains a small number of genes (78) and most genes are involved in male development^7^. In contrast, the X chromosome contains ≈2000 genes with key biological functions^8^, including the largest number of immune-related genes in the human genome and several genes with key roles in airway biology (e.g. the IL-13 receptor)^8^. To prevent excessive gene activity in X-linked genes, one of the X chromosomes must be inactivated in females^6,9^. This process entails global methylation of the X chromosome selected for inactivation (Xi), theoretically resulting in complete gene dosage compensation of X-linked genes^9^. However, there is increasing evidence demonstrating that the methylation of the Xi is a complex process that varies with developmental stage, chronological age and cell or tissue type^10–12^. At least 15% of X-linked genes completely escape inactivation and many others have variable X-methylation and inactivation status^10–15^. As a result, females have higher gene dosage (enhanced expression) of some X-linked genes, which may lead to important biological sex-related differences in different cell systems and across life-span^16^. For instance the excessive expression of X-linked immune genes in females is thought to modify their immune responses conferring overall decreased risk against infections but higher risk for autoimmune conditions^8^. However, our understanding of how methylation of X-linked genes may affect airway disease risk from the beginning of life is incomplete.

The goal of our study was to characterize sex-based epigenomic signatures in the human airway during early life with a particular focus on defining DNA methylation patterns of the X chromosome. To this end, we compared male versus female genome-wide DNA methylation data in nasal airway samples from newborns and infants aged 1–6 months. We also contrasted sex-based human airway DNA methylation patterns in infants born full-term and infants born extremely premature (24 weeks gestation) to examine the potential effect of the intrauterine environment in the development of sex-based airway methylation marks. The latter is particularly important given the strong sex-related differences in the respiratory morbidity of premature infants^1^. Airway epigenomic marks in male and female infants were first examined with a machine learning approach that categorized X-linked genes in two separate groups (with and without sex-based differential methylation patterns) using a principal component (PCA)-based binary classifier and the Matthews Correlation Coefficient, (MCC), which is a comprehensive measure of how good is the agreement between observed and predicted binary classification^17,18^. We then filtered genes without sex-based differential methylation based on predicted biological function and expression in the human airway epithelium.

The impact of our current work is that it provides new insights about the genetically pre-determined sex-based signatures of the airways at birth (DNA methylation patterns). This is clinically relevant because sex-based differences in immune and/or remodeling responses to environmental risk factors (e.g. viral infections and tobacco smoke) may protect or predispose to many chronic pulmonary conditions that begin in early life^19^. Accordingly, characterization of early airway methylation signatures may help us understand sex-based disparities in human respiratory disorders and may ultimately lead to better personalized diagnostic and therapeutic approaches for both sexes.

## Results

### Sex-dependent nasal airway DNA epigenomic methylation signatures in early life

**Figure 1:**
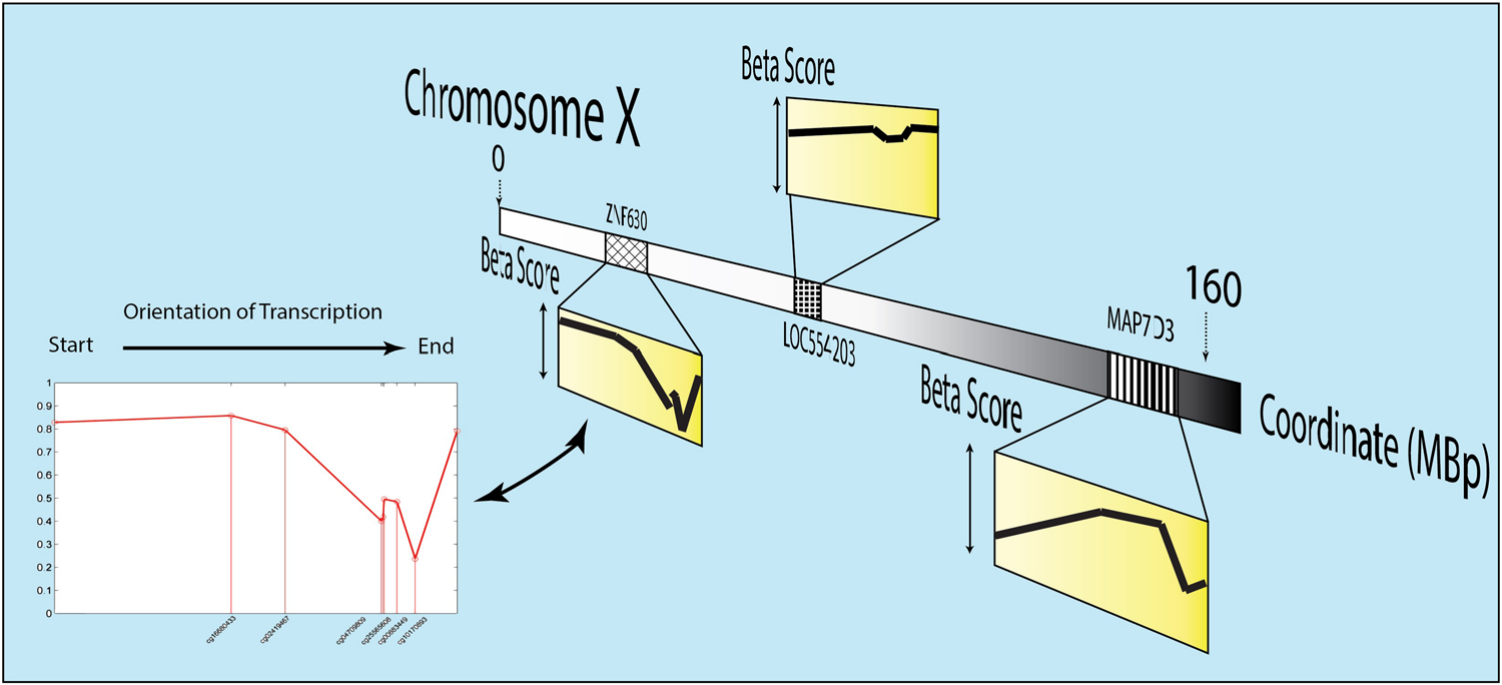
Schematic of Sex-based DNA methylation Signal Acquisition. Multiple CpG methylation values mapped to the same gene were organized according to physical Chromosomal coordinates to create a matrix of patterns (Gene Methylation Signal).

**Figure 2:**
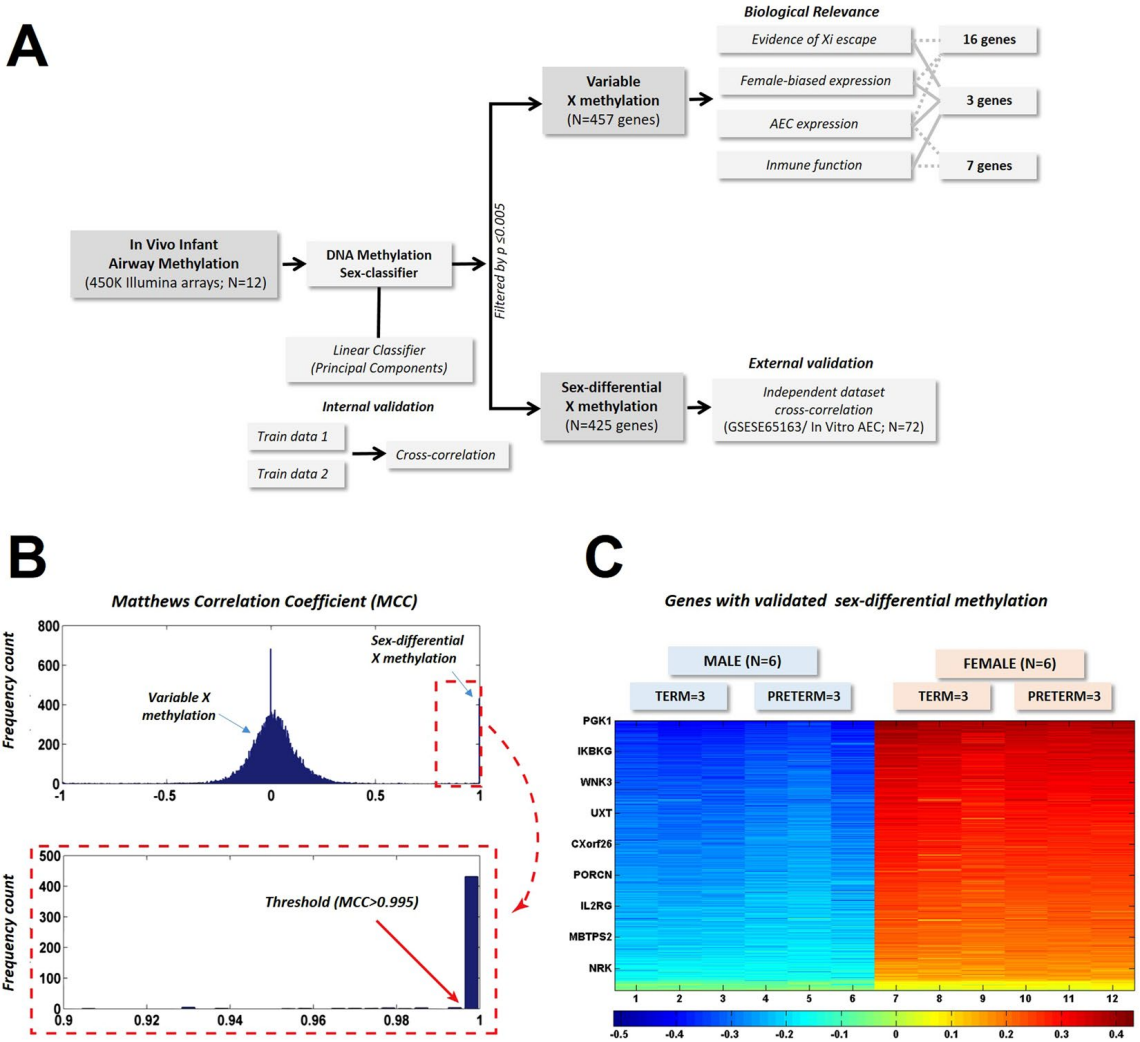
Work Flow of Study. (**A**) Nasal airway genes were divided into those with sex-differential or variable X-chromosome methylation. (**B**) Histogram of the Matthews Correlation Coefficients shows a discernible cluster of X-linked genes above 0.995 (zoom on image). (**C**) Heatmap showing within-group similarity (clustering) of subjects (male or female) but not within term and preterm (n = 425 genes). Color key represents eigen-projections of each methylation pattern (per gene) on the PCA feature corresponding to the largest eigenvalue on the zero-mean, normalized methylation patterns.

To investigate sex-dependent epigenetic differences in the human airways during early life we determined the genome-wide DNA methylation profiles of nasal samples obtained from a group of newborns and infants (N = 12; Supplementary Table 1). For this study we designed a robust sex classification algorithm to generate DNA methylation signatures based on the signal of multiple CpG sites mapped to the same gene instead of comparing single CpG data points (Fig. 1). We estimated experimentally the Matthews Correlation Coefficient (MCC) measure when using principal component analysis (PCA) as a kernel based classifier, followed by an algorithm that performed an exhaustive cross-validation (Fig. 2A). We then analyzed the distribution of the MCC in classification to determine the cut-off point for differential sex-based methylation. As shown in the histogram of the MCC (Fig. 2B), we identified a discernible group of X-linked genes with a MCC > 0.995 using all CpG data points. Of note, when we restricted the classifying algorithm to CpG sites in gene promoters, regardless of the features (PCA projections, average methylation, etc.), we did not observe such clear bimodal distributions (Supplementary Fig. 1). Using this MCC cut-off value, our machine learning algorithm identified 425 distinct genes differentially methylated in the chromosome X of males versus females (Fig. 2). These genes represented ≈50% of the unique protein coding transcripts in the X chromosome with reported CpG sites in the HM450 array (891 genes). After exploring the entire human genome, we found only 1 gene with differential sex-based methylation located in an autosome (*FAM35A* in the chromosome 10). The complete list of genes with significantly different sex-based DNA methylation signatures is presented in the Supplementary Table 2.

To examine the effect of preterm birth in sex-dependent nasal airway DNA methylation signatures, we also analyzed our data according to gestational age classifying infants as full-term (N = 6) or premature (N = 6). As shown in the heatmap of Fig. 2C, we observed that sex-based DNA methylation signatures are essentially unaffected by extreme preterm birth. The latter indicates that the epigenomic modification of the X-chromosome in the airways occurs during early pregnancy and is overall preserved at birth independently of gestational age.

### External validation of sex-dependent nasal airway DNA methylation signatures in early life with pediatric human nasal airway epithelium

To validate our airway, sex-based, DNA methylation signatures (identified in nasal washes) we used an independent population comprised of pediatric nasal airway epithelial samples (GSE65163)^20^. This dataset was retrieved from the GEO database (http://www.ncbi.nlm.nih.gov/geo/). The GSE65163 is a study of genomic DNA methylation patterns and gene expression in African American children aged 10–12 years of age with persistent atopic asthma (n = 36) versus healthy controls (n = 36)^20^. This study of nasal epithelial cultures was relevant to cross-review our observations seen on nasal washes samples from infants that could have contained other cell types (e.g. immune cells). The enrichment of nasal airway epithelial cells (AEC) in the validation dataset was confirmed by ensuring that all samples had at least 80% of ciliated epithelial cells visualized in slides from nasal brushings^20^. In addition, all nasal specimens had expression of the FOXJ1 gene, a specific marker of airway ciliated cells^20^, confirming that the samples represented AEC rather than immune cells. In this analysis we identified that 213 genes had sex-based epigenomics marks in our validation cohort of nasal AEC and that 190 were are present in both populations (nasal washes and nasal AEC). The gene with the largest difference between males and females, based on DNA methylation marks, was *PGK1* followed by other genes mapped in the X-chromosome (e.g. *CHST7, ASFMR1, FMR1*). The top 25 nasal airway genes with most significantly different DNA methylation patterns between males and females after this external cross examination are listed in Table 1. Using gene expression data from the same validation cohort^20^, we found that 15 of the top 25 genes with most significantly different sex-based DNA methylation in both datasets were expressed in the nasal airway epithelium of children^20^. Similar expression levels of these 15 genes were observed in males and females (Supplementary Table 3).

**Table 1:** Top 25 genes with significant sex-based DNA methylation in the nasal airway of infants and children.

Gene	Coordinate	Discovery Dataset (N = 12)	Validation Dataset (N = 72)
		PCA Ratio	PCA Ratio
*PGK1*	77245617	0.558272177	0.726072379
*CHST7*	46317714	0.451207234	0.272775351
*ASFMR1*	146800600	0.431249912	0.159308627
*FMR1*	146800600	0.431249912	0.159308627
*CXorf42*	119262549	0.42492609	0.070609049
*LONRF3*	117991568	0.41294454	0.120282378
*BRCC3*	153952468	0.412234904	0.158846518
*LOC100133957*	47401812	0.397013227	0.15116961
*HTATSF1*	135406459	0.393213575	0.105284092
*MST4*	130984129	0.392315414	0.193206124
*LOC401588*	46289472	0.373302546	0.144862054
*FAM104B*	55186689	0.373139389	0.044954829
*PQBP1*	48639144	0.369732812	0.180396379
*GAB3*	153559641	0.36907422	0.201463872
*OTUD5*	48664552	0.365507365	0.326526154
*ZCCHC12*	117841517	0.356850648	0.222881078
*GLA*	100545822	0.351001143	0.126436623
*KIF4A*	69425734	0.346604603	0.124683181
*SPACA5*	47747921	0.344858624	0.388032883
*SLC9A6*	134894867	0.341351248	0.127843025
*TIMM17B*	48635688	0.340443412	0.180396379
*ZCCHC18*	103242790	0.337535797	0.133992323
*RP2*	46581030	0.3369494	0.09295638

Genes listed according to principal components analysis (PCA) eigenvalue ratios for discovery and validation cohorts.

### Variable X-chromosome DNA methylation in the nasal airway of infants

**Figure 3:**
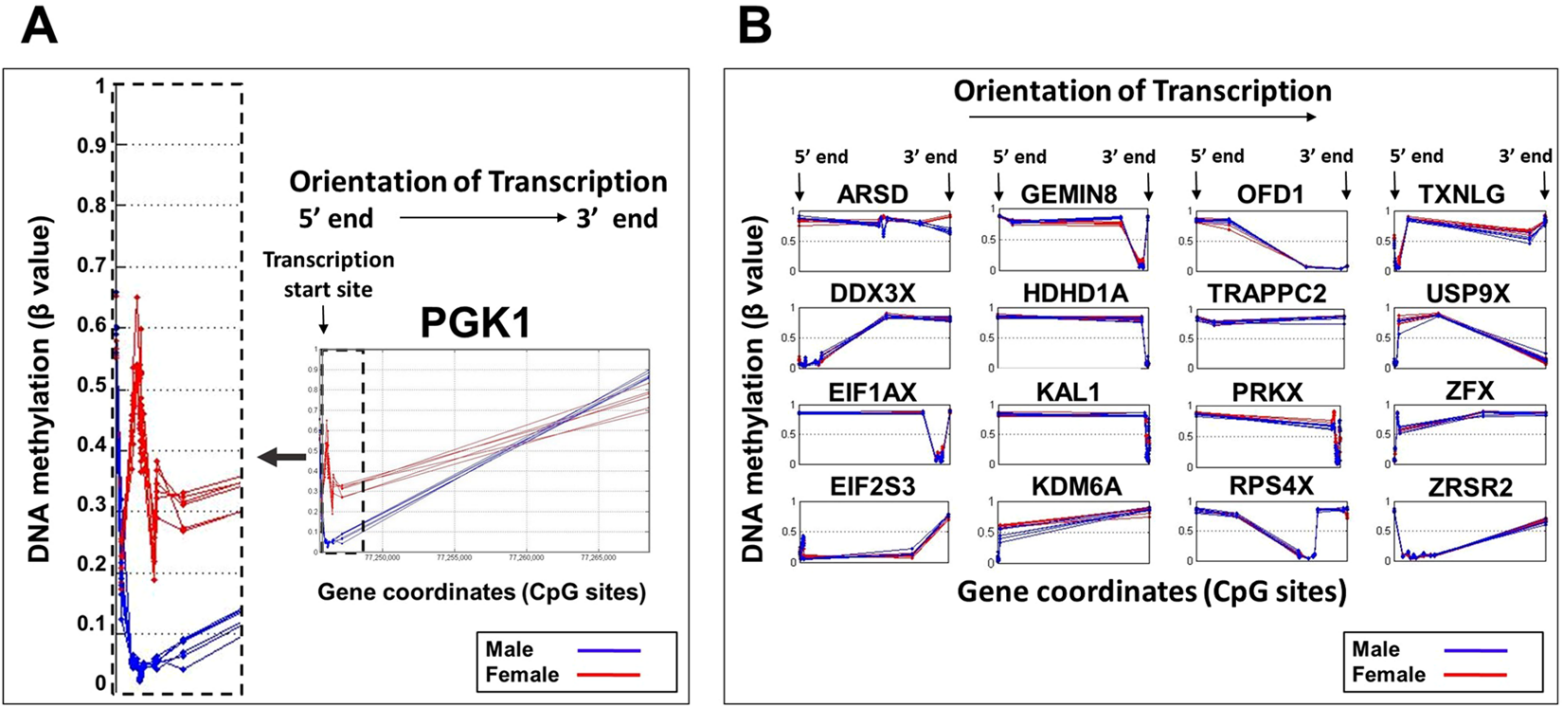
Sex-based DNA methylation signals in X inactivated and Xi-escape genes. Visualization of Gene Methylation Signals for: (**A**) *PGK1*positive control for X-inactivation (sex-differential signal highlighted by zoom on image) and (**B**) X-linked airway escape genes with reported female-biased expression (no sex-differential signal). Data represent 12 human nasal airway infant samples (male N = 6 and female N = 6).

**Figure 4:**
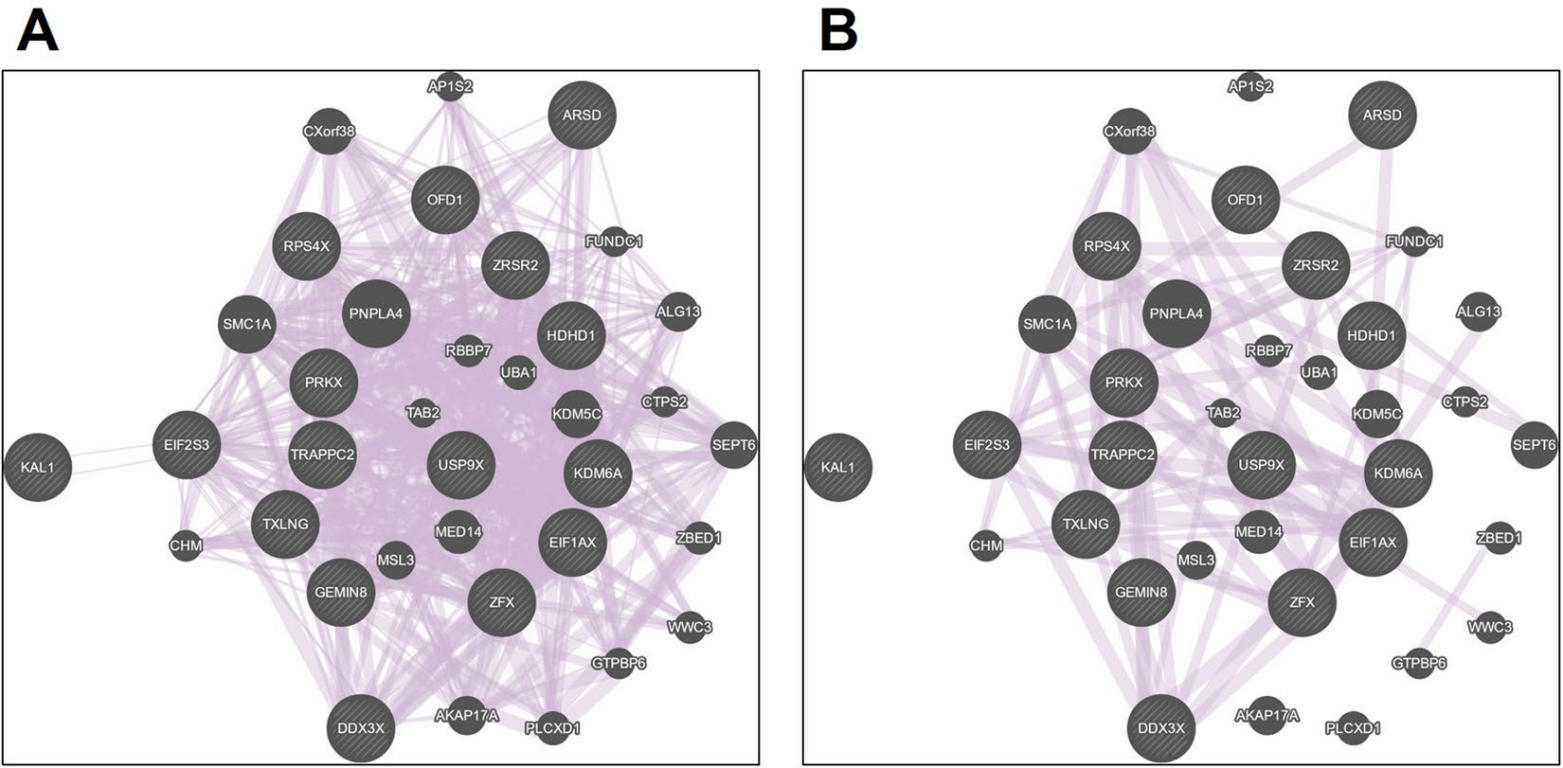
GenMANIA Networks of X-linked Genes with Female-biased Airway Expression. (**A**) Overall overlap with available genomics data and (**B**) Top network corresponding to the COPDGene study dataset (GSE42057).

The variability of X-chromosome inactivation is influenced by species, age/development and cell/tissue type^10–12^, and thus far have not been characterized in the human airways during early life. Accordingly, we next examined DNA methylation signature patterns in genes without significantly different sex-based methylation signal according to our robust sex classification algorithm (Figs 1 and 2). Out of 891 unique protein coding transcripts examined in the X chromosome, we identified 457 genes with potential variability of X-chromosome inactivation (Fig. 2). We filtered these genes based on experimental evidence of escaping X inactivation status and known female-biased expression in 4 different datasets of human lungs (N = 1,395; GSE30219, GSE31210, GSE37745, and GSE41271)^16^. Fig. 3 shows DNA methylation signals of *PGK1* (control X inactivated human gene; Fig. 3A) and the 16 genes selected given that they had confirmed X-linked escape status with reported increased gene transcription in the lungs of adult females across 4 independent cohorts (*ARSD, DDX3X*, *EIF1AX, EIF2S3, GEMIN8, HDHD1, KAL1, KDM6A, OFD1, PRKX, RPS4X, TRAPPC2, TXLNG, USP9X, ZFX, ZRSR2*)^16^. Notably, all 16 analyzed genes had remarkably similar nasal DNA methylation signals in males and female infants (Fig. 3B), which is compatible with escape from X-chromosome inactivation and potential increased transcription in females. The latter is in contrast with our observations in *PGK1* that confirmed differential DNA methylation signals (dose compensation) over the X- chromosomes in males and females (Fig. 3A). Network analysis of these 16 X-linked human airway genes performed with GeneMANIA, (http://genemania.org/)^21^, to import and match interaction networks from public databases of overlapping genes in a set of study subjects or experimental conditions. A strength of this approach is that the top network has a corresponding study, which may be useful to understand the potential relevance of the findings^21^. These analyses showed overall 96.35% overlap with published studies (Fig. 4A). Interestingly, the top network identified (22,78% overlap with 278,447 interactions from GEO; Fig. 4B) was derived from the COPDGene study, one of the largest gene expression studies in individuals with chronic obstructive pulmonary disease (COPD). (GSE42057 dataset)^22^.

### Nasal airway X-chromosome DNA methylation of immune genes

An intriguing feature of the X-chromosome is its enrichment for immune related genes, some of which play key roles in the development of respiratory conditions (e.g. TLR 8/7 and IL-13 receptor)^8^. This observation led us to conduct additional X-chromosome DNA methylation analyses to better define sex-based epigenomic patterns of immune genes in the nasal airway of infants. For these analysis we filtered by biological function 457 X-linked genes without significantly different sex-based methylation signals (genes with variability of X-chromosome inactivation; Fig. 2). To accomplish this we constructed a list of X-linked immune genes (Supplementary Table 4) compiling information from two large Gene Ontology databases: PANTHER (Protein ANalysis THrough Evolutionary Relationships; http://www.pantherdb.org/)^23^ and IRIS (Immunogenetic Related Information Source)^24^. We also included X-linked genes associated with primary immunodeficiencies and/or immune dysregulation in humans^25,26^. This analysis identified 54 genes with variable X-chromosome inactivation with reported immune function. From this filtered list of X-linked immune genes we found 16 with published experimental evidence of escape genes or heterogeneous X-chromosome methylation across species and/or cells (Table 2). Gene Ontology classified several of these X-linked immune genes as “cancer/testis” (cancer antigens) or as genes with primary function and/or expression outside of the respiratory tract (Table 2). We identified 7 immune genes with reported gene expression in the airways. This list of genes included 3 with confirmed female-biased expression in the airways *(DDX3X*, *PRKX, TXLNG*)^16^ and 4 additional immune genes with previous experimental evidence of airway gene expression but without confirmed sex-based differences (*L1CAM, VEGFD, TIMP1, TLR7*).

**Table 2:** X-linked genes with immune function and non-significant sex-based DNA methylation in the nasal airway of infants and children.

Gene	Gene name	Xi status	Aec	Primary site
***DDX3X***	**DEAD-box helicase 3, X-linked**	**Escape**	**Yes**	**Ubiquitous**
***PRKX***	**cAMP-dependent protein kinase catalytic subunit**	**Escape**	**Yes**	**Ubiquitous**
***TXLNG***	**Gamma-taxilin**	**Escape**	**Yes**	**Ubiquitous**
*L1CAM*	Neural cell adhesion molecule L1	Escape	Yes	Neurons, immune cells
*VEGFD*	Vascular endothelial growth factor D	Heterogeneous	Yes	Ubiquitous
*TIMP1*	Metalloproteinase inhibitor 1	Heterogeneous	Yes	Ubiquitous
*TLR7*	Toll-like receptor 7	Heterogeneous	Yes	Ubiquitous
*BTK*	Tyrosine-protein kinase BTK	Heterogeneous	N/A	Lymphocytes
*BMP15*	Bone morphogenetic protein 15	Heterogeneous	N/A	Testis
*DDX53*	DEAD-box helicase 53, X-linked	Heterogeneous	N/A	Testis
*AKAP4*	A-kinase anchor protein 4	Heterogeneous	N/A	Cancer/Testis
*SRPX2*	Sushi repeat-containing protein	Heterogeneous	N/A	Ubiquitous
*GRPR*	Gastrin-releasing peptide receptor	Heterogeneous	N/A	Ubiquitous
*CD40LG*	CD40 ligand	Heterogeneous	N/A	Ubiquitous
*CTAG2*	Cancer/testis antigen 2	Heterogeneous	N/A	Cancer/Testis
*CXorf36*	Deleted in autism-related protein 1	Heterogeneous	N/A	Neurons, ovary

Genes listed according to X-inhibition (Xi) status, expression in airway epithelial cells (AEC) and primary site of expression/function. Genes in bold have prior evidence of escape Xi or heterogeneous X-chromosome methylation and reported expression in AEC.

**Figure 5:**
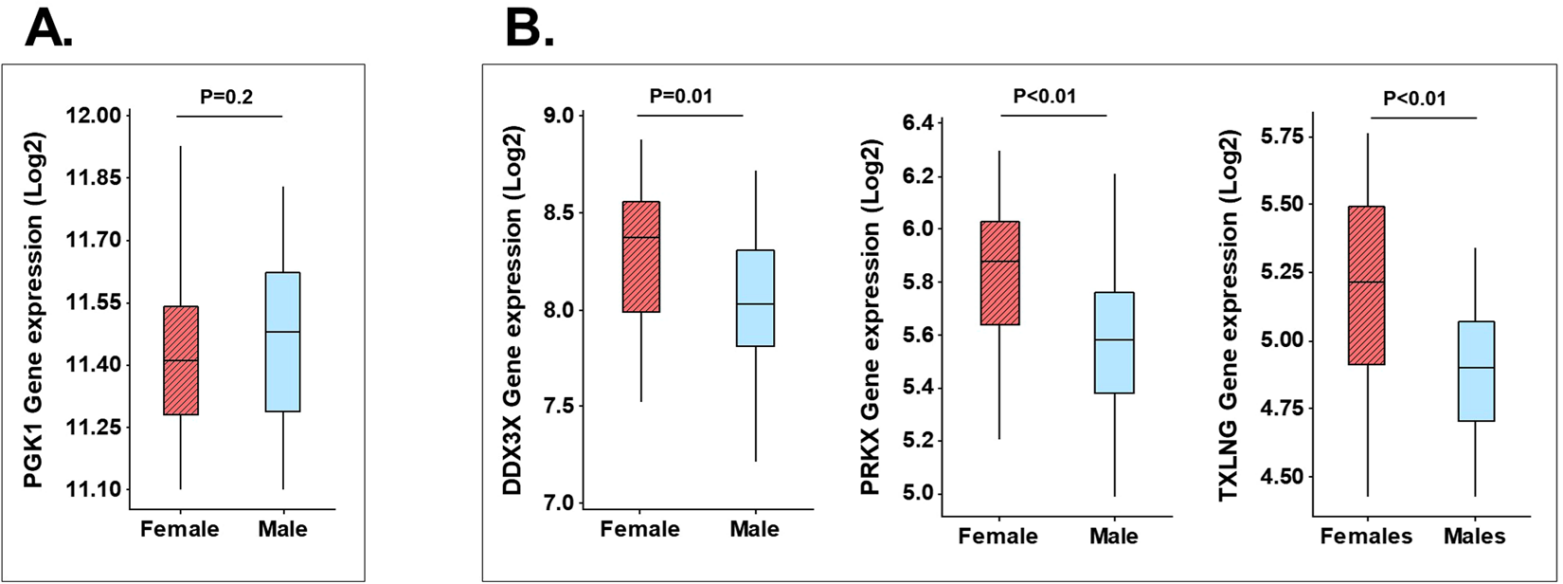
Gene expression of X inactivated and Xi-escape transcripts in pediatric airway epithelium. Nasal airway epithlial cell gene expression (GSE65205)^20^ of female (shaded red box; N = 34) and male (clear blue box; N = 36) children for: (**A**) *PGK1* positive control for X-inactivation (sex-differential methylation), (**B**) *DDX3X*, *PRKX, TXLNG* which are X-linked airway immune escape genes (without sex-differential methylation pattern). Data represent boxplots of mRNA log2 values (Agilent Human Gene Expression arrays; GSE65205)^20^. P value obtained by Wilcoxon rank test.

To explore the potential functional relevance of unbalanced X-chromosome dosage in the pediatric nasal epithelium we next examined gene expression of nasal AEC samples of children included in our validation cohort (GSE65205)^20^. As shown in Fig. 5A we found that PGK1, the gene with the most divergent sex-based DNA methylation signal in our analysis (Fig. 3A), had very similar AEC gene expression in males vs. females suggesting balanced X-chromosome dosage. In contrast, we found that the 3 immune X-linked genes without sex-specific methylation patterns and reported female-biased airway expression in adults had increased expression in the pediatric nasal airway epithelium of females (DDX3X, PRKX, TXLNG, Fig. 5B). The other 4 X-linked immune genes were either not expressed in pediatric nasal epithelial samples (VEGFD and L1CAM) or did not show sex-based differential gene expression (TIMP1 and TLR7).

## Discussion

There is no doubt that in humans respiratory conditions are largely influenced by the individual’s sex resulting in overall higher risk for males than females, particularly during early life^1–5^. Although hormonal, anatomical and behavioral differences are postulated to play a role, these sex-based respiratory differences are already present at birth^1^, suggesting a strong genetic component. Nonetheless, the genetic differences in the airways of males and females during early life have been remarkably understudied and are largely unknown. To address this critical gap, in this study we have used signal processing and machine-learning classification approaches to identify DNA methylation patterns of the X-chromosome and thus identify sex-based DNA methylation epigenomic signatures in the airways of newborns and infants. The impact of this work is that early airway epigenomic marks may reflect initial developmental and/or environmental factors that determine sex-based respiratory phenotypes and overall predisposition to develop respiratory disorders later in life.

To investigate epigenetic differences in the airways of males and females during early life we used genome-wide DNA methylation arrays (Illumina HM450). Bioinformatics analysis of this type of epigenomic data commonly includes the determination of statistical differences at single CpG sites, considering them independent of each other and adjusting for false discovery rate^27^. However, analyzing DNA methylation signals across all CpG sites of each gene may identify otherwise unnoticed biological processes and may offer the foundation for a more robust detector/classifier. Accordingly, to develop a robust sex-classifier for our study, we considered gene methylation as a pattern arranging gene-specific β values according to their physical Chromosomal coordinate (Fig. 1). We feel that our strategy is particularly well-suited to identify sex-based epigenomic signatures in humans given inherent sex group differences due to global methylation (inactivation) of one of the X chromosomes in females^6^. In this scenario, rather than identifying single CpG-site statistical differences between males and females, it becomes more important to detail these differences in individual gene-specific methylation signals (based on multiple CpG sites mapped to the same gene). After interrogating the entire human genome, we identified that virtually all genes with sex-based differential methylation patterns in the airways are located in the X chromosome, with the exception of *FAM35A*, which is located in an autosome (chromosome 10). These results confirm that the X-chromosome contains crucial information about sex-related genetic differences between males and females in different tissues including the airways^8,16^.

Our approach to characterize sex-based DNA methylation epigenomic signatures in the airways of newborns and infants continued with the classification of X-linked genes in two groups: genes with *significant sex-based X methylation* and genes with *variable X methylation* (Fig. 2). In order to distinguish the two groups, we designed a sex-based classifier using PCA and the cross-correlation matrix between patterns followed by an exhaustive cross-validation that yield an accurate likelihood that a gene methylation pattern could be classified by sex (>99.5%). Our findings were cross examined with an independent population of children with nasal epigenomic data available^20^. Notably, although the initial airway sex-based gene signature was obtained in nasal washes (mixed epithelial and airway cell population), it was then positively validated against nasal airway epithelial cell cultures of children. These studies indicate that the X-linked epigenomic marks are present in the human airway epithelium, however, these X-linked marks are likely not airway-specific and may exist ubiquitously or in many other tissues. The importance of identifying these X-linked epigenomic marks in the nasal airway epithelium is that it is the primary cellular interface with the environment and contains crucial epigenetic marks that regulate gene expression in pediatric asthma^20^. Using our sex classification algorithm, we identified *PGK1* as the gene with the most divergent methylation signal in the airways of males versus females in both populations. As visualized in Fig. 3, *PGK1* was not only different between sex groups, its DNA methylation pattern was almost identical in each of the individuals of the same sex (Fig. 3A). This robust sex-based DNA methylation signal may reflect an evolutionary conserved pattern of gene methylation to maintain equal gene expression of *PGK1* in the airway of males and females. In support of this, *PGK1* is often used as positive control gene for X-chromosome inactivation^28^ and “housekeeping gene” due to stable gene expression across individuals in airway epithelial cells^29^. *PGK1* has a vital role activating glycolysis during hypoxia^30^, function that may underlie its conserved methylation pattern in the airway epithelium of males and females during early life.

There is increasing evidence demonstrating that X-chromosome methylation is variable and over 15% of genes can completely escape X-chromosome inactivation^10–15^. These genes, known as “escape genes”, typically have increased transcription in females due to biallelic expression^10–15^. Other X-linked genes have been reported to have variable expression in different species or cell types and may change its X-inactivation pattern during early developmental stages and with aging^10–15^. Accordingly, variable X-chromosome inactivation is currently investigated as a potential genetic mechanism to explain the effect of gender as a risk factor for many conditions including respiratory disorders and infections (both more common in males) as well as autoimmune disorders (more common in females)^8^. Our current airway epigenomic data and sex-related methylation signal analyses support the notion that variable X-chromosome inactivation is a common occurrence. Indeed, we found that in the nasal airway of infants only ≈50% of genes (425 of 891 unique protein coding genes) had robustly different methylation patterns indicative of X inactivation and equal gene expression in males and females. As shown in Fig. 2, these genes formed a tight cluster around MCC > 0.995 based on our DNA methylation sex-classifier; the rest of genes had a variable (nearly normally distributed) MCC measure in the classification as sex-based methylation (Fig. 2). Based on these findings we believe that other X-linked genes in the infant airway may have variable sex-based DNA methylation and some may completely escape X-inactivation leading to enhanced expression in females. To begin to explore this notion we examined a sub-set of 16 well-known X-linked escape genes with reported increased gene transcription in the lungs of adult females across 4 independent cohorts (N = 1,395)^16^. Relative to *PGK1* (positive control for X-chromosome inactivation in the airway), we found that these X-linked airway escape genes had essentially identical DNA methylation signal patterns in males and females (Fig. 3). The potential relevance of these genes in respiratory disease is suggested by our GeneMANIA network analysis^21^ showing the COPDGene study as the top dataset (Fig. 4)^22^. In addition, some of these female-biased genes have functions that predict an important role in the airways. Of particular interest is *DDX3X*, a crucial regulator of the TBK1/IRF3 signaling leading to induction of IFN-β^31,32^, which may potentially modulate airway epithelial innate responses against viral infections.

The X chromosome is known to contain the largest number of immune-related genes of the whole human genome^8^. For this reason, we decided to examine another sub-set of X-linked escape genes with reported airway gene expression and top canonical immune functions. In this list we found 3X-linked escape genes with confirmed female-biased airway expression *(DDX3X*, *PRKX,TXLNG)*^16^ and identified other 4 with reported gene expression in the airway epithelium (*VEGFD, TIMP1, L1CAM, TLR7*)^16^. Visualization of the DNA methylation signal of these genes showed similar patterns in males and females suggesting unbalanced X-chromosome dosage (Fig. 4C). We found that the 3 immune X-linked genes without sex-specific methylation and reported female-biased airway expression in adults (*DDX3X*, *PRKX, TXLNG*)^16^ had increased transcription in the pediatric nasal airway epithelium of females (Fig. 5). Nonetheless, it is important to emphasize that the functional relevance of our findings needs to be further investigated. In fact, our data suggest that sex-based methylation patterns do not always reflect X-linked gene expression differences in the airway epithelium. For instance, we did not identify sex-based differential gene expression in *TIMP1* or *TLR7*, and we observed that two genes with differential sex-based methylation had mildly increased AEC expression in females (*LONRF3, ZCCHC18*; Supplementary Table 3; P < 0.05). Together, these data indicate that the mechanisms mediating sex-biased gene expression in the human AEC are complex and likely not fully explained by differences in DNA methylation patterns.

The presence of X-linked immune escape genes due to non-differential sex-based methylation in the pediatric nasal epithelium (*DDX3X*, *PRKX, TXLNG)* might suggest that females have different airway immune responses, however, there is no clear experimental evidence of this to our knowledge. Conversely, sex differences in immune responses have been reported in other cell systems. For instance, TLR7 ligands induce higher IFNα production in plasmacytoid dendritic cells of females^33^. Moreover, several studies have reported that females have enhanced innate and cellular immune responses across species^34^. Notwithstanding this evidence, the biological basis of immune sex-based differences in humans maybe be multifactorial and whether this is mainly due to unbalanced X-chromosome dosage still requires systematic investigation. In this regard, we believe that future research should address the potential functional role of X-linked escape immune genes with confirmed female-biased airway expression in humans^16^. Here we have established that these genes lack appropriate sex-based differential methylation patterns in the airways of newborns and infants (Fig. 2), and thus may contribute to sex-related differences in the airways from the beginning of life. These early sex-based respiratory phenotypes may impact the development of asthma and other chronic respiratory disorders that originate during childhood^19^.

It is important to clarify the purpose and limitations of our current study. Our machine learning algorithm was only developed to identify X-linked genes with significant differences in methylation patterns. We did not aim to improve performance relative to traditional methods that take into account the potential effect of DNA methylation in gene expression, but we wanted the best possible identification of genes with significant differences. For the purpose of binary classification, we observed that using a PCA-based pattern classifier of methylation across the entire gene yielded a clear bimodal distribution with two separate groups of genes (with and without sex-based differential methylation, Fig. 2B), which was not present when we restricted the classifying algorithm to CpG sites in gene promoters. (Supplementary Figure 1) Although differences in the methylation patterns of males vs. females were better visualized examining the entire gene rather than promoter sites alone, it is important to emphasize that CpG sites in gene promoters regions are most likely to influence gene expression, thus the analysis presented should not be extrapolated to epigenomic studies aimed to investigate the functional implications of DNA methylation –as opposed to the binary sex-based classification of X-linked genes presented here-. In addition, given that our machine learning algorithm was trained only with X-linked genes it may not be suitable to identify genes with sex-specific DNA methylation on autosomes.

In summary, our study provides new evidence that sex-based epigenomic signatures due to variable X-chromosome DNA methylation are already present in the airways at birth, which might be relevant for the development of respiratory conditions later in life. Elucidating the genetic basis of sex differences of the respiratory system may help guide personalized therapy to predict, prevent and treat respiratory disorders in both sexes from childhood to adulthood.

## Methods

### Study population and Nasal sampling

Nasal washes were collected from newborns and infants aged 1–6 months admitted to the Children’s National Health System in Washington D.C. for non-respiratory reasons (e.g. diagnostic or surgical procedures). To examine the effect of gestational age in sex-related methylation we included a subset of nasal samples from very premature children (born 24–25 weeks gestational age) obtained prior to discharge from the neonatal intensive care unit at Children’s National Health System. Supplementary Table 1 shows baseline characteristics of all subjects included in the study (N = 12). We used a standard nasal lavage technique consisting of gently washing the nasal cavity with 3–4 mL of sterile normal saline as previously described^35^. This study was approved by the Institutional Review Board of Children’s National Medical Center, Washington, DC, informed consent was obtained from all participants and all experiments were performed in accordance with relevant guidelines and regulations.

### DNA methylation profiling

DNA methylation profiling was conducted in the Genomics Core of the Children’s Research institute (Children’s National Medical Center) using Illumina’s Infinium Human Methylation 450k (HM450) BeadChip arrays (Illumina, San Diego, CA) to interrogate more than 450,000 methylation sites within and outside of CpG islands. After extraction and purification from nasal samples, the obtained DNA was treated with bisulfite and hybridized to HM450 BeadArrays following the manufacturer’s guidelines. The DNA was labeled with a fluorescent dye and then scanned using an Illumina BeadArray Reader. Sequencing data was analyzed using the reference sequence of the human reference genome Hg18. Some transcripts are presented as common synonyms identified in literature search and databases referenced for biological relevance. Control normalization and background subtraction was conducted using Illumina’s algorithm to generate β-values. The methylation status of each CpG site was measured as the ratio of signal from methylated probe to the sum of both methylated and unmethylated signals (β value, ranges from 0, unmethylated, to 1, fully methylated).

### General approach to analyze DNA methylation signals

To identify sex-based methylation signatures we developed a novel algorithm to account for widespread methylation differences between males and females. Rather than using the assumption of independence or weak correlation between single CpG sites, we analyzed methylation as a gene-specific *signal*, that is, by arranging β methylation values in an ordered sequence (according to their Chromosomal coordinate) we associated a short methylation *pattern* to every gene. We started by arranging the methylation data as arrays of *signals* grouped by genes as shown in Fig. 1. For every set of readings, we selected the CpG sites associated with a specific gene to create a matrix of patterns. All CpG sites were included in the determination of the methylation pattern taking into account their coordinates and beta score without further adjustments. The initial dataset contained the information about the individual’s sex, useful for external testing. We evaluated quantitatively the significance of our findings with experimental statistics (see below) that provided us with an objective score to study our data. In the context of this work, the terms *signal* and *pattern* are used rather interchangeably.

### Computational Analyses

#### Principal component analysis (PCA) classifier

All data processing was performed using the commercial software package (MATLAB R2016, The MathWorks Inc., Natick, MA). The data obtained from the DNA methylation signals was first processed gene by gene, with an unsupervised, principal component analysis (PCA) classifier, which was based on the matrix of cross-correlation coefficients between patterns^17^. PCA may be performed by eigenvalue decomposition (EVD) of a data cross-correlation matrix or by singular value decomposition (SVD) of a raw data matrix. We chose the former approach by convenience but numerical results are identical to the SVD case. When effective, the unsupervised classifier was able to separate clear clusters, with zero error in the classification (when comparing vs the ground truth) by only using the two largest PCA projections. The ratio between the largest and second largest eigenvalues of the classification kernel (matrix) was used to rank all genes. This ratio is a measure of dissimilarity between classes in the set of patterns^17,18^. In order to select which genes had indeed significantly different patterns between genders, the pattern matrix was divided into two subsets, one for training and another one for further external testing of a supervised classifier, which follows the same linear classifier based on PCA of the signals^17,18^. The procedure of training and external testing was then repeated a number of times in an exhaustive cross-validation fashion.

#### Matthews Correlation Coefficient (MCC) of classification

When all the iterations were completed, a simple statistic, MCC of classification was calculated^17,18^. MCC is essentially used as a measure of the quality of a binary (two-class) classification. It takes into account true and false positives and negatives, which achieves a rather balanced measure that takes into account all inputs of the confusion matrix. By definition

1 $$Mcc=\frac{TP\ast TN-FP\ast FN}{\sqrt[2]{(TP+FP)(TP+FN)(TN+FP)(TN+FN)}}$$

Where TP is the number of true positives, TN the number of true negatives, FP the number of false positives and FN the number of false negatives. The MCC may be seen as a correlation coefficient between the observed and predicted binary classifications, as the coefficient gets closer to 1, it represents a near perfect prediction. Values around 0 show the classifier performs as a random predictor (worst case) and values close to −1 indicate a total disagreement between prediction and observation, or somehow a consistent error in classification. To calculate MCC of classification in our study, at each iteration we selected training and testing sets from all the available combinations, which allowed us to calculate a relatively high number of independent evaluations even in a small number of samples. For instance, by following this rule, in an experiment with only six samples in two classes we could attain $$N=2\times 3\times {(\begin{array}{c}6\\ 3\end{array})}^{2}=2400$$ tests, in our sample estimate of MCC per gene methylation pattern. Based on the distribution of MCC values in our dataset (Fig. 2) we filtered the gene list at MCC > 0.995, which selected the genetic methylation patterns that can be classified by sex with a balanced combination of accuracy, sensitivity, specificity and recall^17,18^ greater than 99.5%. These genes were considered to have a *sex-specific DNA methylation pattern* (significantly different between male and female). The remainder genes were considered to have *variable sex-based methylation*.

## Electronic supplementary material

Supplementary information
