# Author Correction: Characterization of Sex-Based Dna Methylation Signatures in the Airways During Early Life

**DOI:** 10.1038/s41598-020-60913-7

**Published:** 2020-02-26

**Authors:** Cesar L. Nino, Geovanny F. Perez, Natalia Isaza, Maria J. Gutierrez, Jose L. Gomez, Gustavo Nino

**Affiliations:** 1 Department of Electronics Engineering, Pontificia Universidad Javeriana, Bogota, Colombia; 2 Division of Pulmonary and Sleep Medicine, Children’s National Medical Center, Washington, DC USA; 3 Department of Pediatrics, George Washington University School of Medicine and Health Sciences, Washington, DC USA; 4 Center for Genetic Medicine, Children’s National Medical Center, Washington, DC USA; 5 Division of Neonatology, Children’s National Medical Center, Washington, DC USA; 6 Division of Pediatric Allergy Immunology, Johns Hopkins University School of Medicine, Baltimore, MD USA; 7 Division of Pulmonary, Critical Care and Sleep Medicine, Yale University School of Medicine, New-Haven, CT USA

Correction to: *Scientific Reports* 10.1038/s41598-018-23063-5, published online 03 April 2018

This Article contains a typographical error in the Results section, under the sub-heading ‘Nasal airway X-chromosome DNA methylation of immune genes’.

“To accomplish this we constructed a list of X-linked immune genes (Supplementary Table 4) compiling information from two large Gene Ontology databases: PANTHER (Protein ANalysis THrough Evolutionary Relationships; http://www.pantherdb.org/)^23^ and IRIS (Immunogenetic Related Information Source)^24^.”

should read:

“To accomplish this we constructed a list of X-linked immune genes compiling information from two large Gene Ontology databases: PANTHER (Protein ANalysis THrough Evolutionary Relationships; http://www.pantherdb.org/)^23^ and IRIS (Immunogenetic Related Information Source)^24^.”

