# Supplementary material for: Characterization of Sex-Based Dna Methylation Signatures in the Airways During Early Life
Source: Sci Rep. 2018 Apr 3;8:5526. doi: 10.1038/s41598-018-23063-5 (PMC5882800; doi:10.1038/s41598-018-23063-5)
Supplement: Supplementary file 1 — Supplementary information [file 41598_2018_23063_MOESM1_ESM.docx]

**Supplementary information**

**CHARACTERIZATION OF SEX-BASED DNA METHYLATION SIGNATURES IN THE AIRWAYS DURING EARLY LIFE**

*Cesar L. Nino, PhD, Geovanny F. Perez, MD, Natalia Isaza, MD, Maria J Gutierrez MD, Jose L Gomez,MD, MSc, Gustavo Nino, MD, MSc*

**Supplementary Table 1**

| **Gender** | **Race/Ethinicity** | **Gestational age (weeks)** | **Chronological age (months)** |
| --- | --- | --- | --- |
| Female | Black/African American | 25 | 1 |
| Female | Black/African American | 24 | 2 |
| Female | Black/African American | 25 | 2 |
| Female | White/Caucasian | 38 | 4 |
| Female | Black/African American | 38 | 5 |
| Female | White/Caucasian | 38 | 6 |
| Male | White/Caucasian | 24 | 2 |
| Male | Black/African American | 23 | 1 |
| Male | Black/African American | 25 | 2 |
| Male | White/Hispanic | 41 | 5 |
| Male | Black/African American | 37 | 3 |
| Male | White/Caucasian | 38 | 6 |

**Supplementary Figure 1**. Histogram of the Matthews Correlation Coefficients using all CpG sites (red) shows a discernible cluster of X-linked genes above 0.995 (dotted square), which is not clearly seen in the histogram generated with only CpG sites in promoter regions (blue).

**
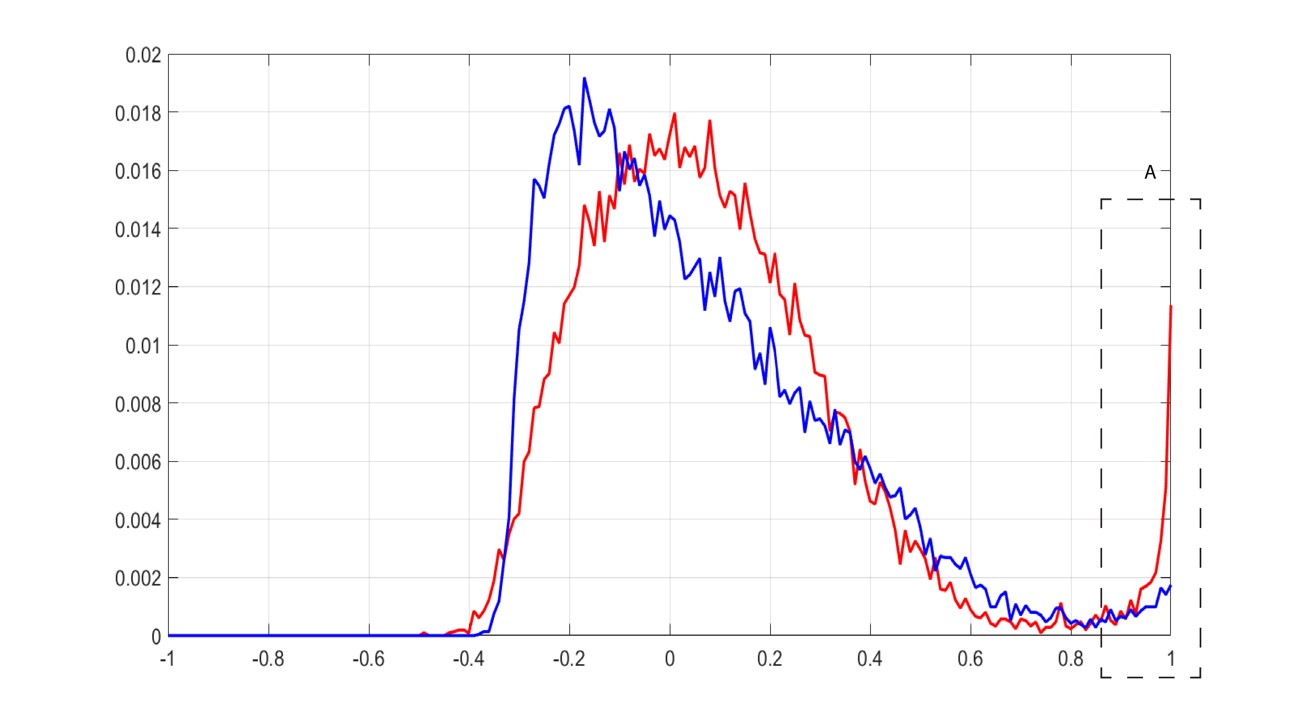
**

**Supplementary Table 2**

| **GENE NAME** | **Coordinate** | **Chromosome** | **MCC** | **Eigenvalue ratio** |
| --- | --- | --- | --- | --- |
| PGK1 | 77245617 | X | 1.000 | 0.558272177 |
| LAS1L | 64649347 | X | 1.000 | 0.45483133 |
| CHST7 | 46317714 | X | 1.000 | 0.451207234 |
| APEX2 | 55042860 | X | 1.000 | 0.436725363 |
| CXorf39 | 103297333 | X | 1.000 | 0.434121937 |
| ASFMR1 | 146800600 | X | 1.000 | 0.431249912 |
| FMR1 | 146800600 | X | 1.000 | 0.431249912 |
| ACSL4 | 108800209 | X | 1.000 | 0.425792545 |
| CXorf42 | 119262549 | X | 1.000 | 0.42492609 |
| CXorf40A | 148429416 | X | 1.000 | 0.414818554 |
| LONRF3 | 117991568 | X | 1.000 | 0.41294454 |
| BRCC3 | 153952468 | X | 1.000 | 0.412234904 |
| HMGB3 | 149901328 | X | 1.000 | 0.40693727 |
| MAGEH1 | 55493879 | X | 1.000 | 0.399335783 |
| LOC100133957 | 47401812 | X | 1.000 | 0.397013227 |
| HTATSF1 | 135406459 | X | 1.000 | 0.393213575 |
| MST4 | 130984129 | X | 1.000 | 0.392315414 |
| ZIC3 | 136474799 | X | 1.000 | 0.388697881 |
| RNF113A | 118889024 | X | 1.000 | 0.378107024 |
| LOC401588 | 46289472 | X | 1.000 | 0.373302546 |
| FAM104B | 55186689 | X | 1.000 | 0.373139389 |
| PQBP1 | 48639144 | X | 1.000 | 0.369732812 |
| GAB3 | 153559641 | X | 1.000 | 0.36907422 |
| CSTF2 | 99961469 | X | 1.000 | 0.365789541 |
| OTUD5 | 48664552 | X | 1.000 | 0.365507365 |
| ZCCHC12 | 117841517 | X | 1.000 | 0.356850648 |
| PIGA | 15248628 | X | 1.000 | 0.35148728 |
| GLA | 100545822 | X | 1.000 | 0.351001143 |
| KIF4A | 69425734 | X | 1.000 | 0.346604603 |
| SPACA5 | 47747921 | X | 1.000 | 0.344858624 |
| FAM120C | 54113838 | X | 1.000 | 0.343918278 |
| MAP7D3 | 135128405 | X | 1.000 | 0.342893722 |
| SLC9A6 | 134894867 | X | 1.000 | 0.341351248 |
| TIMM17B | 48635688 | X | 1.000 | 0.340443412 |
| ZCCHC18 | 103242790 | X | 1.000 | 0.337535797 |
| RP2 | 46581030 | X | 1.000 | 0.3369494 |
| ZC4H2 | 64053606 | X | 1.000 | 0.336258992 |
| SMARCA1 | 128459042 | X | 1.000 | 0.333168256 |
| HPRT1 | 133421336 | X | 1.000 | 0.332967102 |
| DOCK11 | 117512699 | X | 1.000 | 0.33039539 |
| TAZ | 153292481 | X | 1.000 | 0.328129957 |
| EDA | 68751331 | X | 1.000 | 0.327912445 |
| CXorf40B | 148852567 | X | 1.000 | 0.327119411 |
| MAP3K7IP3 | 30786865 | X | 1.000 | 0.325657644 |
| BEX4 | 102356376 | X | 1.000 | 0.324721023 |
| PRPS2 | 12719077 | X | 1.000 | 0.323667536 |
| ATP6AP1 | 153309017 | X | 1.000 | 0.321524853 |
| DNASE1L1 | 153283524 | X | 1.000 | 0.319915963 |
| IKBKG | 153423239 | X | 1.000 | 0.313801695 |
| PSMD10 | 107217714 | X | 1.000 | 0.313154241 |
| FAM127A | 133993620 | X | 1.000 | 0.31056848 |
| GPR101 | 135940487 | X | 1.000 | 0.309984937 |
| SLC10A3 | 153369856 | X | 1.000 | 0.308576151 |
| PDK3 | 24392767 | X | 1.000 | 0.307962901 |
| ZNF449 | 134305256 | X | 1.000 | 0.306665309 |
| WDR44 | 117363381 | X | 1.000 | 0.303830125 |
| SCML1 | 17664732 | X | 1.000 | 0.303707786 |
| LAGE3 | 153358611 | X | 1.000 | 0.30240641 |
| ZNF711 | 84384525 | X | 1.000 | 0.300351291 |
| OCRL | 128501681 | X | 1.000 | 0.299244841 |
| C1GALT1C1 | 119644052 | X | 1.000 | 0.299060404 |
| SAT1 | 23709696 | X | 1.000 | 0.297880344 |
| MID1IP1 | 38545210 | X | 1.000 | 0.295321625 |
| TAF9B | 77272729 | X | 1.000 | 0.294186429 |
| GNL3L | 54573055 | X | 1.000 | 0.291547838 |
| RPL36A | 100531661 | X | 1.000 | 0.291486521 |
| SCML2 | 18167898 | X | 1.000 | 0.291194193 |
| NDUFB11 | 46886629 | X | 1.000 | 0.291069618 |
| USP51 | 55529804 | X | 1.000 | 0.290560671 |
| PGRMC1 | 118253599 | X | 1.000 | 0.28991664 |
| NCRNA00086 | 134383206 | X | 1.000 | 0.289891713 |
| RAP2C | 131174907 | X | 1.000 | 0.28797308 |
| DGKK | 50227969 | X | 1.000 | 0.287407474 |
| ARMCX3 | 100764119 | X | 1.000 | 0.284298854 |
| PCDH19 | 99437525 | X | 1.000 | 0.281370675 |
| FAM70A | 119277712 | X | 1.000 | 0.281347471 |
| VBP1 | 154097342 | X | 1.000 | 0.279886971 |
| STAG2 | 122921297 | X | 1.000 | 0.279291766 |
| NDUFA1 | 118888320 | X | 1.000 | 0.27825403 |
| ZBTB33 | 119268412 | X | 1.000 | 0.278136349 |
| ZNF673 | 46191055 | X | 1.000 | 0.278010362 |
| G6PD | 153413333 | X | 1.000 | 0.278006765 |
| MOSPD2 | 14800922 | X | 1.000 | 0.277892216 |
| RPL10 | 153278979 | X | 1.000 | 0.27771598 |
| ATG4A | 107220194 | X | 1.000 | 0.27544848 |
| ARHGEF9 | 62773087 | X | 1.000 | 0.274701957 |
| BRWD3 | 79947816 | X | 1.000 | 0.2735357 |
| TSPAN7 | 38305270 | X | 1.000 | 0.273034497 |
| APLN | 128612606 | X | 1.000 | 0.272834512 |
| MSL3 | 11684703 | X | 1.000 | 0.272114888 |
| CXorf57 | 105741608 | X | 1.000 | 0.271645011 |
| SYP | 48933009 | X | 1.000 | 0.271535238 |
| NR0B1 | 30232975 | X | 1.000 | 0.270360201 |
| VMA21 | 150315479 | X | 1.000 | 0.269691963 |
| PDZD11 | 69423143 | X | 1.000 | 0.267904407 |
| PHF6 | 133334390 | X | 1.000 | 0.267824315 |
| BEX5 | 101295762 | X | 1.000 | 0.267020269 |
| LOC158572 | 49528338 | X | 1.000 | 0.262962408 |
| WNK3 | 54317688 | X | 1.000 | 0.261683876 |
| RBMX2 | 129363146 | X | 1.000 | 0.260641145 |
| SLC9A7 | 46499386 | X | 1.000 | 0.260357189 |
| ZNF41 | 47190866 | X | 1.000 | 0.257555229 |
| HNRNPH2 | 100549431 | X | 1.000 | 0.257145936 |
| FAM127B | 134012935 | X | 1.000 | 0.257085551 |
| RLIM | 73750590 | X | 1.000 | 0.256357683 |
| RAB33A | 129132550 | X | 1.000 | 0.255884842 |
| YIPF6 | 67635024 | X | 1.000 | 0.255775801 |
| FANCB | 14777515 | X | 1.000 | 0.253367076 |
| EMD | 153259529 | X | 1.000 | 0.251047775 |
| USP27X | 49530171 | X | 1.000 | 0.250421511 |
| IRAK1 | 152930154 | X | 1.000 | 0.250300749 |
| PABPC1L2A | 72214316 | X | 1.000 | 0.249611265 |
| MTCP1 | 153946584 | X | 1.000 | 0.247920969 |
| XIAP | 122821100 | X | 1.000 | 0.247373227 |
| FAM155B | 68640395 | X | 1.000 | 0.246943016 |
| MAOA | 43398858 | X | 1.000 | 0.242790111 |
| GPRASP1 | 101792015 | X | 1.000 | 0.24227975 |
| CXorf58 | 23835375 | X | 1.000 | 0.241718571 |
| PRPS1 | 106757916 | X | 1.000 | 0.24161365 |
| TBC1D8B | 105931500 | X | 1.000 | 0.241491836 |
| LOC100272228 | 148856680 | X | 1.000 | 0.239737799 |
| IL13RA1 | 117744817 | X | 1.000 | 0.238674855 |
| TMEM47 | 34557761 | X | 1.000 | 0.238045338 |
| PLP2 | 48913718 | X | 1.000 | 0.238032753 |
| SLC25A43 | 118416440 | X | 1.000 | 0.237763912 |
| LAMP2 | 119455542 | X | 1.000 | 0.237241819 |
| SPIN3 | 57034540 | X | 1.000 | 0.237115186 |
| INGX | 70628170 | X | 1.000 | 0.233098316 |
| ARHGAP4 | 152841954 | X | 1.000 | 0.232800515 |
| CHRDL1 | 109804318 | X | 1.000 | 0.232071746 |
| TCEAL1 | 102770206 | X | 1.000 | 0.230222418 |
| HDX | 83643510 | X | 1.000 | 0.229763896 |
| PABPC1L2B | 72140113 | X | 1.000 | 0.2296458 |
| RPS6KA3 | 20078473 | X | 1.000 | 0.229445549 |
| FOXO4 | 70232180 | X | 1.000 | 0.228148343 |
| UPF3B | 118867348 | X | 1.000 | 0.227299224 |
| UPRT | 74410077 | X | 1.000 | 0.226140666 |
| LDOC1 | 140098167 | X | 1.000 | 0.226139941 |
| NCRNA00087 | 134057177 | X | 1.000 | 0.222900603 |
| WDR45 | 48819066 | X | 1.000 | 0.222234856 |
| ZNF75D | 134247562 | X | 1.000 | 0.221566148 |
| NKAP | 118958798 | X | 1.000 | 0.220849762 |
| SLC25A14 | 129300253 | X | 1.000 | 0.22035989 |
| ZNF182 | 47720165 | X | 1.000 | 0.218155157 |
| MID2 | 106955406 | X | 1.000 | 0.2177854 |
| NUP62CL | 106267140 | X | 1.000 | 0.21707145 |
| PRDX4 | 23594813 | X | 1.000 | 0.216725686 |
| GRIPAP1 | 48718551 | X | 1.000 | 0.216350096 |
| TIMM8A | 100488238 | X | 1.000 | 0.215786679 |
| UXT | 47398138 | X | 1.000 | 0.214325672 |
| AFF2 | 147389254 | X | 1.000 | 0.214248951 |
| RPGR | 38029717 | X | 1.000 | 0.214226107 |
| EFNB1 | 67964245 | X | 1.000 | 0.213729903 |
| DACH2 | 85289251 | X | 1.000 | 0.213282638 |
| RRAGB | 55760631 | X | 1.000 | 0.213165716 |
| CETN2 | 151746657 | X | 1.000 | 0.212590809 |
| TSR2 | 54483153 | X | 1.000 | 0.212271681 |
| ZNF81 | 47580733 | X | 1.000 | 0.209678394 |
| MTCP1NB | 153943374 | X | 1.000 | 0.209218418 |
| TMEM185A | 148517124 | X | 1.000 | 0.208061022 |
| KLHL34 | 21583851 | X | 1.000 | 0.20720356 |
| GPRASP2 | 101853054 | X | 1.000 | 0.205403825 |
| ZMYM3 | 70376882 | X | 1.000 | 0.204869806 |
| GPC3 | 132921516 | X | 1.000 | 0.203075041 |
| ZDHHC9 | 128766958 | X | 1.000 | 0.202907622 |
| PRAF2 | 48815813 | X | 1.000 | 0.20268826 |
| TMLHE | 154422823 | X | 1.000 | 0.201617011 |
| ANKRD58 | 118775710 | X | 1.000 | 0.201380368 |
| CHIC1 | 72698458 | X | 1.000 | 0.199784957 |
| MED12 | 70254360 | X | 1.000 | 0.199695916 |
| SLC35A2 | 48645656 | X | 1.000 | 0.198997617 |
| HCCS | 11038649 | X | 1.000 | 0.198305072 |
| TFE3 | 48784459 | X | 1.000 | 0.198300458 |
| IDH3G | 152704561 | X | 1.000 | 0.197768898 |
| ACOT9 | 23631864 | X | 1.000 | 0.196452572 |
| ZXDA | 57950815 | X | 1.000 | 0.19634048 |
| TCEAL8 | 102395509 | X | 1.000 | 0.194131195 |
| SSR4 | 152712054 | X | 1.000 | 0.194059615 |
| TCEAL2 | 101266634 | X | 1.000 | 0.192089034 |
| ARMCX2 | 100797318 | X | 1.000 | 0.191561644 |
| TBC1D25 | 48282464 | X | 1.000 | 0.191080557 |
| LOC100131434 | 148427347 | X | 1.000 | 0.19089301 |
| LOC139201 | 72697188 | X | 1.000 | 0.190114145 |
| CNKSR2 | 21301738 | X | 1.000 | 0.189030971 |
| ELK1 | 47380945 | X | 1.000 | 0.189005158 |
| SMS | 21868337 | X | 1.000 | 0.187517704 |
| DDX26B | 134481213 | X | 1.000 | 0.187255733 |
| UBE2A | 118591941 | X | 1.000 | 0.187135874 |
| SLC25A5 | 118484960 | X | 1.000 | 0.187095509 |
| MCTS1 | 119620460 | X | 1.000 | 0.186875268 |
| SLC16A2 | 73556534 | X | 1.000 | 0.18603676 |
| PIM2 | 48657789 | X | 1.000 | 0.185739044 |
| CFP | 47370764 | X | 1.000 | 0.18490198 |
| CD99L2 | 149686562 | X | 1.000 | 0.183724859 |
| GPC4 | 132263198 | X | 1.000 | 0.182899279 |
| ARMCX6 | 100758259 | X | 1.000 | 0.182058143 |
| FAM58A | 152506946 | X | 1.000 | 0.181512536 |
| ARX | 24931855 | X | 1.000 | 0.181480254 |
| DUSP9 | 152559861 | X | 1.000 | 0.181203614 |
| CXorf26 | 75308090 | X | 1.000 | 0.181089458 |
| TMEM164 | 109131981 | X | 1.000 | 0.180542162 |
| ELF4 | 129027290 | X | 1.000 | 0.18039382 |
| DYNLT3 | 37584575 | X | 1.000 | 0.179587665 |
| CXorf22 | 35847538 | X | 1.000 | 0.179528837 |
| MPP1 | 153660588 | X | 1.000 | 0.179000648 |
| SLITRK2 | 144705941 | X | 1.000 | 0.178925077 |
| APOOL | 84144488 | X | 1.000 | 0.178901437 |
| PCSK1N | 48574561 | X | 1.000 | 0.177931565 |
| NKRF | 118606720 | X | 1.000 | 0.176442089 |
| RGAG4 | 71264131 | X | 1.000 | 0.176329898 |
| FUNDC2 | 153908008 | X | 1.000 | 0.175756126 |
| UTP14A | 128866439 | X | 1.000 | 0.174546378 |
| NHS | 17302719 | X | 1.000 | 0.173822679 |
| ARAF | 47305123 | X | 1.000 | 0.173700818 |
| ABCB7 | 74292520 | X | 1.000 | 0.173445358 |
| ZXDB | 57634195 | X | 1.000 | 0.173015946 |
| WAS | 48426359 | X | 1.000 | 0.171432657 |
| SRPX | 37893850 | X | 1.000 | 0.171338995 |
| PHKA1 | 71716736 | X | 1.000 | 0.170164109 |
| GRIA3 | 122144345 | X | 1.000 | 0.169353344 |
| HSD17B10 | 53474968 | X | 1.000 | 0.169081851 |
| MOSPD1 | 133850520 | X | 1.000 | 0.168628293 |
| PHF8 | 53981612 | X | 1.000 | 0.168567554 |
| MCART6 | 103232278 | X | 1.000 | 0.1684097 |
| APOO | 23761387 | X | 1.000 | 0.168027559 |
| TSPYL2 | 53127841 | X | 1.000 | 0.16801033 |
| RBM41 | 106246111 | X | 1.000 | 0.16748514 |
| FGF13 | 137542118 | X | 1.000 | 0.166787449 |
| BCORL1 | 128943097 | X | 1.000 | 0.166478668 |
| FAM127C | 133982907 | X | 1.000 | 0.164970957 |
| BEX1 | 102204452 | X | 1.000 | 0.164749923 |
| CCDC160 | 133197244 | X | 1.000 | 0.164268176 |
| IRS4 | 107862409 | X | 1.000 | 0.164231943 |
| ESX1 | 103381485 | X | 1.000 | 0.163783269 |
| THOC2 | 122564265 | X | 1.000 | 0.161894161 |
| TRMT2B | 100151633 | X | 1.000 | 0.161844177 |
| ARMCX5 | 101740132 | X | 1.000 | 0.160912647 |
| MAGT1 | 76970815 | X | 1.000 | 0.159469908 |
| FAM123B | 63326239 | X | 1.000 | 0.158153845 |
| RNF128 | 105822541 | X | 1.000 | 0.157267974 |
| TAF1 | 70502270 | X | 1.000 | 0.157256892 |
| SASH3 | 128741259 | X | 1.000 | 0.157145114 |
| RAI2 | 17728550 | X | 1.000 | 0.157077802 |
| ARHGEF6 | 135577065 | X | 1.000 | 0.15701972 |
| GABRQ | 151556555 | X | 1.000 | 0.156924374 |
| LANCL3 | 37314963 | X | 1.000 | 0.155866867 |
| BHLHB9 | 101861937 | X | 1.000 | 0.15536006 |
| LOC100303728 | 118484151 | X | 1.000 | 0.15528623 |
| LOC729609 | 19918285 | X | 1.000 | 0.155225776 |
| PPP1R3F | 49011763 | X | 1.000 | 0.154973023 |
| RBM3 | 48317223 | X | 1.000 | 0.154799187 |
| KLF8 | 56274838 | X | 1.000 | 0.154598344 |
| PORCN | 48251768 | X | 1.000 | 0.151654073 |
| ERCC6L | 71349988 | X | 1.000 | 0.150069382 |
| ENOX2 | 129586163 | X | 1.000 | 0.148758624 |
| KIAA2022 | 73877467 | X | 1.000 | 0.148286256 |
| AIFM1 | 129097741 | X | 1.000 | 0.148116007 |
| PJA1 | 68297744 | X | 1.000 | 0.148082334 |
| EBP | 48264760 | X | 1.000 | 0.148050269 |
| MIR718 | 152938816 | X | 1.000 | 0.146326835 |
| SHROOM4 | 50354293 | X | 1.000 | 0.146245109 |
| CXorf65 | 70240468 | X | 1.000 | 0.146056506 |
| PHKA2 | 18820724 | X | 1.000 | 0.145816922 |
| TMSB15B | 103103221 | X | 1.000 | 0.145491129 |
| XKRX | 100055610 | X | 1.000 | 0.144963198 |
| TSC22D3 | 106843821 | X | 1.000 | 0.144708503 |
| MAP3K15 | 19288555 | X | 1.000 | 0.144475242 |
| RPL39 | 118805407 | X | 1.000 | 0.144273723 |
| UBL4A | 153365260 | X | 1.000 | 0.143467397 |
| TSPAN6 | 99771477 | X | 1.000 | 0.142368325 |
| TMSB4X | 12902605 | X | 1.000 | 0.140739414 |
| CASK | 41259803 | X | 1.000 | 0.140302336 |
| FAM122C | 133757027 | X | 1.000 | 0.140007914 |
| NSDHL | 151748838 | X | 1.000 | 0.139264429 |
| IGBP1 | 69269700 | X | 1.000 | 0.13851977 |
| MORF4L2 | 102817198 | X | 1.000 | 0.137172952 |
| CENPI | 100240009 | X | 1.000 | 0.136778127 |
| POU3F4 | 82649003 | X | 1.000 | 0.135044747 |
| TMSL3 | 12902605 | X | 1.000 | 0.133872039 |
| XK | 37429376 | X | 1.000 | 0.132744873 |
| CUL4B | 119542886 | X | 1.000 | 0.131795757 |
| MAGIX | 48904847 | X | 1.000 | 0.130356026 |
| PTCHD1 | 23261602 | X | 1.000 | 0.129616223 |
| SNORD61 | 135789155 | X | 1.000 | 0.129234541 |
| ATRX | 76718863 | X | 1.000 | 0.128792747 |
| MORC4 | 106115060 | X | 1.000 | 0.128191721 |
| MECP2 | 152941024 | X | 1.000 | 0.127816275 |
| OGT | 70668146 | X | 1.000 | 0.127814844 |
| FTSJ1 | 48219184 | X | 1.000 | 0.126995431 |
| FLJ30058 | 130019469 | X | 1.000 | 0.126433618 |
| RBM10 | 46888104 | X | 1.000 | 0.12593281 |
| POLA1 | 24621395 | X | 1.000 | 0.125122562 |
| MTMR8 | 63529751 | X | 1.000 | 0.124759937 |
| NUDT11 | 51250117 | X | 1.000 | 0.124244904 |
| PIN4 | 71317734 | X | 1.000 | 0.123635193 |
| COL4A6 | 107286720 | X | 1.000 | 0.122594525 |
| LOC92249 | 62564521 | X | 1.000 | 0.122551018 |
| ARMCX1 | 100690692 | X | 1.000 | 0.122463221 |
| GPR50 | 150094370 | X | 1.000 | 0.122245669 |
| ABCD1 | 152642075 | X | 1.000 | 0.122146834 |
| MAGED1 | 51562399 | X | 1.000 | 0.12139646 |
| NHSL2 | 71047263 | X | 1.000 | 0.121045766 |
| HMGN5 | 80263153 | X | 1.000 | 0.120740462 |
| IL2RG | 70244231 | X | 1.000 | 0.120683644 |
| SH3BGRL | 80343671 | X | 1.000 | 0.120403907 |
| DCAF12L2 | 125126214 | X | 1.000 | 0.11790756 |
| MTM1 | 149487111 | X | 1.000 | 0.117298372 |
| SYTL4 | 99816750 | X | 1.000 | 0.117056725 |
| MAGEE2 | 74919637 | X | 1.000 | 0.117033151 |
| GSPT2 | 51502229 | X | 1.000 | 0.116862392 |
| MTMR1 | 149612184 | X | 1.000 | 0.1149239 |
| TMSB15A | 101656990 | X | 1.000 | 0.113843725 |
| FAM3A | 153387666 | X | 1.000 | 0.113388633 |
| SLITRK4 | 142543690 | X | 1.000 | 0.113323501 |
| IDS | 148384930 | X | 1.000 | 0.113101985 |
| SUV39H1 | 48439129 | X | 1.000 | 0.113100349 |
| PRRG1 | 37093032 | X | 1.000 | 0.113093127 |
| CCDC22 | 48977433 | X | 1.000 | 0.113074515 |
| MBNL3 | 131336193 | X | 1.000 | 0.112985236 |
| ZNF674 | 46289472 | X | 1.000 | 0.112852892 |
| 6-Sep | 118634301 | X | 1.000 | 0.112139308 |
| LOC349408 | 12834105 | X | 1.000 | 0.112049104 |
| TLR8 | 12834105 | X | 1.000 | 0.112049104 |
| TCEAL6 | 101281873 | X | 1.000 | 0.112014837 |
| NGFRAP1 | 102516526 | X | 1.000 | 0.109874748 |
| DLG3 | 69580311 | X | 1.000 | 0.109750076 |
| HDAC8 | 71466570 | X | 1.000 | 0.109265079 |
| PHF16 | 46656098 | X | 1.000 | 0.108705854 |
| ZDHHC15 | 74507477 | X | 1.000 | 0.107567797 |
| DKC1 | 153643672 | X | 1.000 | 0.104689459 |
| TMEM187 | 152889964 | X | 1.000 | 0.104161701 |
| MSN | 64803043 | X | 1.000 | 0.102619552 |
| PAK3 | 110073549 | X | 1.000 | 0.10233145 |
| GK | 30580069 | X | 1.000 | 0.101917838 |
| TCEAL3 | 102748112 | X | 1.000 | 0.101873848 |
| RBMX | 135780706 | X | 1.000 | 0.101715207 |
| HS6ST2 | 131589894 | X | 1.000 | 0.101497303 |
| NUDT10 | 51090808 | X | 1.000 | 0.101150502 |
| GPKOW | 48863085 | X | 1.000 | 0.101059983 |
| SNORA70 | 153280467 | X | 1.000 | 0.100591153 |
| SYN1 | 47316351 | X | 1.000 | 0.100308587 |
| RPS6KA6 | 83328989 | X | 1.000 | 0.10025097 |
| MMGT1 | 134873290 | X | 1.000 | 0.100170157 |
| BEX2 | 102451098 | X | 1.000 | 0.099583008 |
| ZNF275 | 152252340 | X | 1.000 | 0.099140364 |
| FLNA | 153230235 | X | 1.000 | 0.098841101 |
| FAM122B | 133755521 | X | 1.000 | 0.098553392 |
| FAM50A | 153324221 | X | 1.000 | 0.097709346 |
| AR | 66679192 | X | 1.000 | 0.097680933 |
| FGD1 | 54489097 | X | 1.000 | 0.097362734 |
| RAB39B | 154141922 | X | 1.000 | 0.096867462 |
| MAGED2 | 54850458 | X | 1.000 | 0.096626522 |
| WBP5 | 102497909 | X | 1.000 | 0.096508874 |
| STARD8 | 67783855 | X | 1.000 | 0.096415692 |
| MBTPS2 | 21766869 | X | 1.000 | 0.096218033 |
| ARMCX4 | 100559393 | X | 1.000 | 0.094834387 |
| ZMAT1 | 101026214 | X | 1.000 | 0.094462245 |
| OPHN1 | 67184125 | X | 1.000 | 0.093527668 |
| BCAP31 | 152619214 | X | 1.000 | 0.093325685 |
| TCEAL5 | 102415831 | X | 1.000 | 0.092398386 |
| GDI1 | 153317669 | X | 1.000 | 0.091930098 |
| NAA10 | 152851059 | X | 1.000 | 0.091814313 |
| IL1RAPL2 | 103696236 | X | 1.000 | 0.091764151 |
| MIR505 | 138834005 | X | 1.000 | 0.090657314 |
| IGSF1 | 130235226 | X | 1.000 | 0.090581493 |
| HUWE1 | 53576873 | X | 1.000 | 0.088785196 |
| SLC7A3 | 70062269 | X | 1.000 | 0.088515175 |
| HDAC6 | 48544568 | X | 1.000 | 0.08830706 |
| ATP6AP2 | 40324637 | X | 1.000 | 0.087992002 |
| USP11 | 46976183 | X | 1.000 | 0.087628435 |
| ZNF280C | 129165855 | X | 1.000 | 0.087066315 |
| PDHA1 | 19271131 | X | 1.000 | 0.084527856 |
| CYSLTR1 | 77469495 | X | 1.000 | 0.084503868 |
| CXorf56 | 118557640 | X | 1.000 | 0.084356552 |
| NONO | 70418880 | X | 1.000 | 0.082724713 |
| REPS2 | 16874262 | X | 1.000 | 0.082100083 |
| SH3KBP1 | 19463403 | X | 1.000 | 0.081286852 |
| RBBP7 | 16773067 | X | 1.000 | 0.079379246 |
| MAGEE1 | 75563840 | X | 1.000 | 0.079065297 |
| TRO | 54963526 | X | 1.000 | 0.078859275 |
| CITED1 | 71438219 | X | 1.000 | 0.078478401 |
| KCNE1L | 108754035 | X | 1.000 | 0.078290141 |
| COL4A5 | 107568393 | X | 1.000 | 0.078282695 |
| GPR34 | 41431788 | X | 1.000 | 0.077457386 |
| UBQLN2 | 56606493 | X | 1.000 | 0.076397346 |
| FHL1 | 135055873 | X | 1.000 | 0.075159854 |
| GLUD2 | 120008771 | X | 1.000 | 0.074069032 |
| AMMECR1 | 109326157 | X | 1.000 | 0.073597689 |
| PCYT1B | 24486290 | X | 1.000 | 0.072301072 |
| CA5B | 15665487 | X | 1.000 | 0.070173435 |
| ITM2A | 78509221 | X | 1.000 | 0.069929869 |
| PNMA3 | 151974746 | X | 1.000 | 0.069661627 |
| MIR542 | 133503099 | X | 1.000 | 0.066221634 |
| KLHL13 | 116916418 | X | 1.000 | 0.066150801 |
| PABPC5 | 90576259 | X | 1.000 | 0.062898589 |
| HAUS7 | 152366462 | X | 1.000 | 0.061274657 |
| GABRE | 150872922 | X | 1.000 | 0.061258821 |
| ARHGAP6 | 11067063 | X | 1.000 | 0.060382803 |
| KLHL15 | 23915277 | X | 1.000 | 0.060306176 |
| ZNF630 | 47810390 | X | 1.000 | 0.059778128 |
| IL1RAPL1 | 28514147 | X | 1.000 | 0.059592062 |
| F8A2 | 153766737 | X | 1.000 | 0.059585032 |
| F8A3 | 153766737 | X | 1.000 | 0.059585032 |
| F8A1 | 153766737 | X | 1.000 | 0.058298868 |
| DCAF12L1 | 125511585 | X | 1.000 | 0.05745214 |
| CLCN5 | 49572516 | X | 1.000 | 0.057396874 |
| CACNA1F | 48970418 | X | 1.000 | 0.057340833 |
| NXT2 | 108665449 | X | 1.000 | 0.056514134 |
| FAM35A | 88843588 | 10 | 1.000 | 0.055980765 |
| BCYRN1 | 70350334 | X | 1.000 | 0.054718083 |
| WDR13 | 48339601 | X | 1.000 | 0.054665971 |
| PIR | 15313038 | X | 1.000 | 0.047548799 |
| CXorf21 | 30487221 | X | 1.000 | 0.044694945 |
| PRICKLE3 | 48918956 | X | 1.000 | 0.044687551 |
| RAB9B | 102965069 | X | 1.000 | 0.041702567 |
| XG | 2725017 | X | 1.000 | 0.038388925 |
| MIR106A | 133132022 | X | 1.000 | 0.023571118 |
| TMEM31 | 102852334 | X | 1.000 | 0.013309677 |
| MIR105-1 | 151311375 | X | 1.000 | 0.011482574 |
| MIR105-2 | 151313568 | X | 1.000 | 0.009741248 |
| VCX2 | 8099190 | X | 1.000 | 0.008718541 |
| AWAT1 | 69371112 | X | 1.000 | 0.006897426 |
| SNORA56 | 153655424 | X | 1.000 | 0.006299017 |
| GAGE12F | 49103989 | X | 1.000 | 0.003606441 |

**Supplementary Table 3**

| X-linked Gene | Male (median Log 2) | Female (median Log 2) | P value |
| --- | --- | --- | --- |
| PGK1 | 11.48 | 11.41 | 0.288 |
| CHST7 | 5.55 | 5.48 | 0.7053 |
| FMR1 | 5.185 | 5.16 | 0.288 |
| LONRF3 | 4.615 | 4.78 | 0.0418 |
| BRCC3 | 8.425 | 8.44 | 0.299 |
| HTATSF1 | 9.12 | 9.12 | 0.3161 |
| PQBP1 | 7.46 | 7.425 | 0.7277 |
| GAB3 | 4.535 | 4.62 | 0.1038 |
| OTUD5 | 9.405 | 9.37 | 0.6225 |
| GLA | 8.875 | 8.8 | 0.4007 |
| KIF4A | 4.615 | 4.74 | 0.0828 |
| SLC9A6 | 6.645 | 6.73 | 0.0746 |
| TIMM17B | 10.94 | 10.87 | 0.7098 |
| ZCCHC18 | 4.81 | 4.85 | 0.0351 |
| RP2 | 6.76 | 6.76 | 0.9569 |
